# Radiation-induced alterations in the cancer microenvironment (Review)

**DOI:** 10.3892/mi.2026.299

**Published:** 2026-01-23

**Authors:** Haiying Liu, Xiaoping Zhu, Zhenxing He, Jianshe Yang

**Affiliations:** 1 Medical College, Hexi University, Zhangye, Gansu 734000, P.R. China; 2 Department of General Surgery, The Third People's Hospital of Longgang District, Shenzhen and Shenzhen Clinical Medical School, Guangdong Pharmaceutical University, Shenzhen, Guangdong 518115, P.R. China; 3 Shanghai Tenth People's Hospital, Tongji University School of Medicine, Shanghai 200072, P.R. China

**Keywords:** tumor microenvironment, radio-biological response, radiotherapy, metabolite, nanotechnology, nanoconfinement

## Abstract

Radiation therapy is routinely used for the treatment of cancer; however, the development of radiation resistance in some tumor cells can lead to poor efficacy. Cancer cells can alter their metabolic status following exposure to radiation to reduce radiation-induced cytotoxicity, ultimately enabling them to survive radiation injury and adapt to the changed environment. It has been indicated that these metabolic changes in cancer cells affect the cancer microenvironment, and certain metabolite changes within the microenvironment may, in turn, promote cancer cell proliferation. Therefore, combining radiotherapy with targeted metabolic treatments may enhance the effectiveness of cancer therapy. The present review discusses the metabolic reprogramming of cancer cells following radiotherapy, and the resulting microenvironmental metabolic characteristics. Furthermore, the present review introduces the novel findings on innate nano-confinements, which may influence the cancer microenvironment by regulating energy metabolism and signaling pathways through new mechanisms.

## 1. Introduction

**Figure 1:**
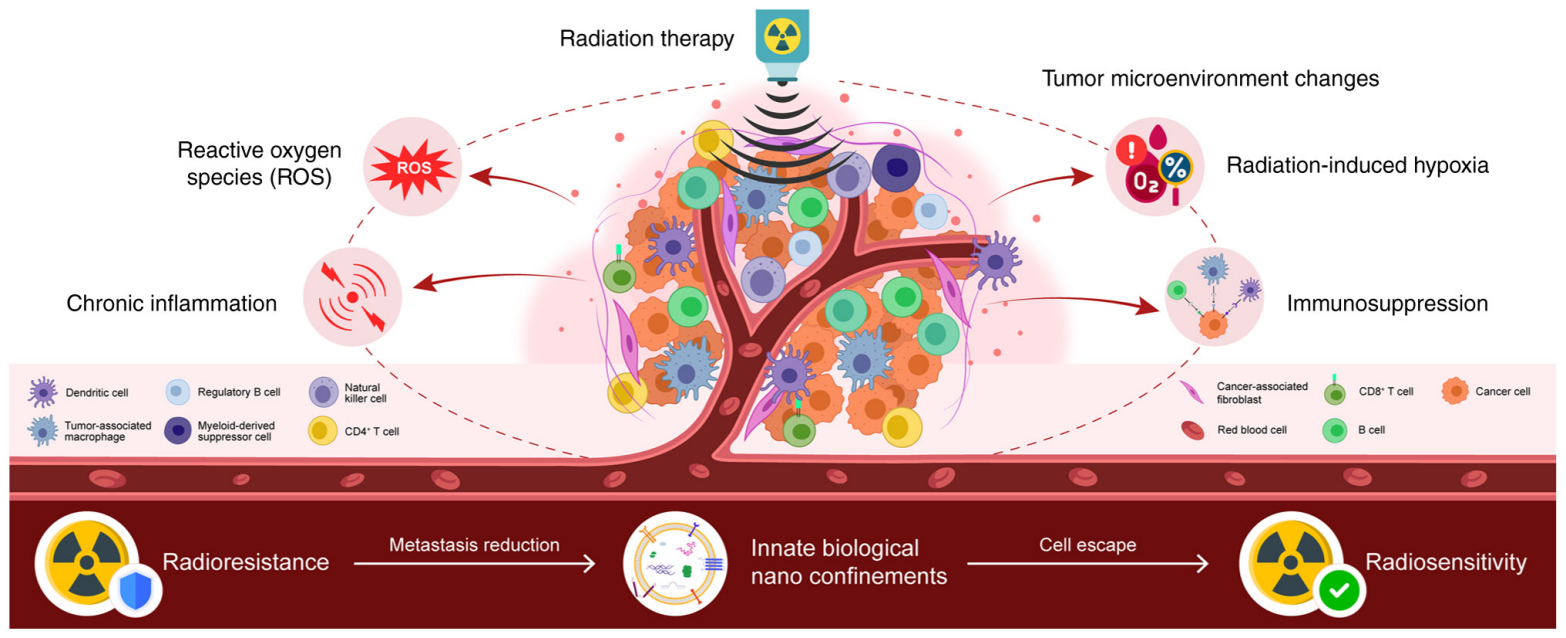
Ionizing radiation induces oxidative stress and metabolic changes, leading to TME alterations in activity of the glycolysis/pentose phosphate pathways, lipid metabolism, and redox homeostasis, which promote cancer cell survival. Novel findings on innate biological nano-confinements may influence the TME via new mechanisms regulating energy metabolism and signaling pathways. TME, tumor microenvironment.

Radiation therapy is a common cancer treatment; however, radioresistance may result in treatment failure (1). Cancer cells can alter their metabolic status following radiation therapy to reduce the cytotoxic effects, thereby diminishing the treatment efficacy (2). The tumor microenvironment (TME) is defined as the cellular and non-cellular components surrounding tumor cells. This includes immune cells and stromal material. As recently reported, altered tumor metabolism influences the TME, and alterations in the metabolite composition of the TME can support tumor growth (3,4). Therefore, targeting tumor metabolism in combination with radiotherapy may enhance the efficacy of cancer treatment (5). The present review discusses the metabolic reprogramming of cancer cells following radiation exposure and summarizes the findings involving the TME to illustrate the metabolic characteristics of radiotherapy. Furthermore, the present review introduces novel findings on innate nanobiological confinements that may influence the TME via novel mechanisms regulating energy metabolism and signaling pathways (Fig. 1).

For the present review, ‘Boolean Operators’ such as AND, OR and NOT were used to search for relevant research articles/reviews published over the past two decades from the PubMed database and Clinical Trials website (https://ClinicalTrials.gov) for cancer microenvironment alterations induced by radiation. Thereafter, only the articles/reviews containing both ‘cancer microenvironment alterations’ and ‘radiation/radiotherapy’ were filtered into the investigational pool.

## 2. Metabolic alterations in the TME following radiotherapy

Radiotherapy is a widely used cancer treatment, with almost half of cancer patients receiving radiation therapy. It is usually administered over a period of days or weeks in a targeted, sub-dose manner, primarily eliminating cancer cells due to DNA injury through the over expressed reactive oxygen species (ROS) (6). Radiotherapy can serve as an adjuvant therapeutic model following radical cancer surgery, or it can serve as a neoadjuvant therapy for advanced-stage cancer. Its combination with chemotherapy can enhance the anticancer effect (7). A number of experimental and clinical studies have explored its combination with immunotherapy (8-10); in addition, investigations are continuing on this matter (Clinical trial no. NCT02223923). Despite considerable positive progress over the past decades, radiation resistance remains a key challenge in the treatment of cancer. The key to overcoming this issue may lie in targeting the TME, specifically the variety of non-malignant cells surrounding cancer cells. Radiation therapy alters the TME, potentially triggering interactions among its components that contribute to radiotherapy resistance. Therefore, a more in-depth exploration of TME interaction mechanisms may be critical, as it may identify mechanisms which can be used to enhance the therapeutic efficacy of radiotherapy.

Radiation-induced vascular damage, hypoxia and chronic inflammation in the TME may promote cancer cell survival and radioresistance (11). Due to endothelial damage, vascular damage and hypoxia also occur, possibly owing to the reduced oxygen consumption in irradiated cells and the survival of certain blood vessels that maintain the oxygen and nutrients to supply to the tumor continuously (12,13). The immune clearance of cancer cells following radiotherapy is crucial for tumor control (14), as the antitumor immune response can be activated by radiation-induced DNA damage and in turn, this increases antigen release from cancer cells (15). However, the co-existence of hypoxia and high levels of ROS can create a chronic inflammatory state, in which CD8^+^ T-cells, CD4^+^ T-cells, natural killer and dendritic cells are suppressed, resulting in the low efficacy of tumor elimination (16). As an additional tumor-promoting condition, the higher numbers of regulatory T-cells (Tregs) and bone marrow-derived suppressor cells (MDSCs) ultimately lead to an immunosuppressive environment.

Cell metabolism is critical for the TME, and metabolic dysregulation can support cancer cell growth and therapeutic resistance, both hallmark features of cancer (17). Metabolites and cytokines secreted by cancer cells can reprogram the metabolism of other TME components, thereby altering its composition and promoting tumor growth (18). This metabolic reprogramming forms a symbiotic association, enabling the exchange of metabolites to meet the needs of different cell types for metabolic within the TME. These interactions provide a potential avenue for targeted cancer treatment without disrupting normal cellular functions. Therefore, studying metabolic changes in the TME in response to radiation is essential for developing novel therapeutic strategies.

Cellular ROS can lead to DNA damage and the destruction of cellular structures, including cell membranes and blood vessels, resulting in transient hypoxia and nutrient deprivation in cancer cells, while simultaneously increasing antitumor immune cell activity in the TME (19,20). Cancer cells reprogram their metabolic state to counteract radiation-induced damage (21). The metabolic changes that promote cancer survival under radiotherapy may involve four main features: First, radiation can enhance the express efficiency of glycolysis pathway (GLP) and the pentose phosphate pathway (PPP), which are highly associated with glycolytic adenosine triphosphate (ATP) synthesis, nucleotide biosynthesis, membrane phospholipid synthesis, and nicotinamide adenine dinucleotide phosphate (NADPH) regeneration to support antioxidant responses (22). Second, elevated ROS can activate novel lethal pathways, for example, neoplastic iron death (23). Furthermore, damaged DNA and cell membranes require metabolic support to facilitate repair processes (24). Finally, cancer-TME metabolic interactions can lead to the insufficient supply of nutrients to the tumor by disrupting local blood vessels (25). The nutrients provided by the surrounding matrix thus become a crucial driving force for cancer cell metabolism (18).

## 3. Reconstruction of glycolysis and the PPP following radiotherapy

In the long-term, the efficacy of radiotherapy is affected by the biological characteristics of the tumor, such as inherent radioresistance, pathological hypoxia and the consequent complexity of the TME. Consequently, the more active biological pathways in cancer cells have been considered potential therapeutic targets. Antibodies and small-molecule inhibitors targeting glycolysis and the PPP have further been demonstrated in preclinical studies with promising results (26-28).

The comprehensive treatment regimen of cetuximab and radiotherapy has been approved by the FDA (29), whereas therapies such as small molecular drugs (erlotinib/lapatinib) are still in the stages of clinical trials (30,31). Cetuximab and panitumumab are classical anti-epidermal growth factor receptor (EGFR) antibodies, which were widely used in the treatment of colorectal cancer. Through an autocrine and paracrine manner, EGFR is activated by members from the EGF family. By combining with these EGFs, EGFR dimers and downstream signaling pathways (KRAS-BRAF-MEK-ERK, PI3K, AKT and STAT) are activated efficiently. As regards the EGFR, its tyrosine phosphorylation level has been demonstrated to be upregulated in growth-restricted squamous cells (32) and breast cancer cells (33). Furthermore, EGFR signaling enhances the expression of hexokinase II (HKII), while inhibiting the expression of pyruvate kinase isoenzyme M2(34). Radiation can further activate the hypoxia-inducing factor 1α subunit, resulting in the increased transcription of several hypoxia-related genes, such as glucose transporter 1 (GLUT1) and lactate dehydrogenase A (35). This upregulation increases glycolysis throughput in glioblastoma (GBM), potentially providing a survival advantage following radiotherapy. Several preclinical trials have explored the combination of radiotherapy with 2-deoxyglucose (2-DG) to counteract this metabolic adaptation (36-38). The PPP mainly provides NADPH for reducing glutathione (GSH) and nucleotides for DNA synthesis (39). This pathway is rapidly upregulated to response to DNA damage, which is caused by oxidative stress and radiation exposure. The GLUT1 receptor also regulates the hexokinase-1 (HKI) and pyruvate kinase (PK) expression level under the stimulation of EGF. HKII has been observed to be overexpressed in various types of cancer cells (40). In the cytoplasm, the voltage-dependent anion channel protein on the mitochondrial membrane has been observed to be specifically bind with HKII to resist glucose-6-phosphate (G-6-P) concentration-dependent feedback inhibition, owing to the high maintenance of G-6-P concentrations in cancer cells following radiation (41). As a biosynthetic substrate, G-6-P links glycolysis to the PPP, providing a rationale for the use of PPP inhibitors in combination with radiotherapy (22). The nicotinamide analog, 6-aminonicotinamide (6-AN), has been shown to inhibit G6PD activity and enhance sensitivity to radiation (42). The combination of radiotherapy with 2-DG and 6-AN considerably improves the efficacy of radiotherapy. Genes involved in the regulation of glycolysis are contributors to cancer cell survival. Radiation exposure can activate HIF1, and increase glycolysis and the level of antioxidants (5). The upregulation of EGF signaling induced by radiation may lead to the accumulation of glycolytic intermediates in cancer cells (43). During glycolysis, G-6-P can be shunted into the PPP, where the ribonucleotides produced are essential for post-radiation DNA repair (44).

## 4. Radiation can alter the redox metabolic response in cancer cells

High energy generated by ionizing radiation drives the formation of ROS through the chemical and physical interaction between ions and cellular components, which mainly includes bioactive hydrogen peroxide (H_2_O_2_), superoxide anion (O^2-^) and hydroxyl radical (OH^-^). Persistent high levels of ROS lead to oxidative stress, which may influence the structural and functional state of DNA, cell or intra-cell membranes, proteins, and so on, ultimately triggering cell death. Although increase levels of ROS may promote cancer growth and progress, excessively high levels of ROS can have anticancer effects (6).

An adaptive change in redox metabolism can be induced by radiation-induced oxidative stress in cancer cells (45,46), and the potential association between radiation and consequently enhanced antioxidant metabolism has been confirmed in previous studies (47,48). The NRF2-mediated antioxidant responses activated by ionizing radiation has been well demonstrated in breast cancer cells (49). Additionally, the analysis of the blood of patients with cancer receiving radiation therapy has revealed reduced levels of the antioxidant, GSH (50), whereas genome-wide metabolic models from The Cancer Genome Atlas have indicated the antioxidant NADPH with an increased synthesis rate in radioresistant cancer cells (51).

## 5. Radiotherapy-induced structural molecule repair relies on metabolism

Radiotherapy, by its ion beams, directly attacks and injures the structures of cellular macromolecular and organelles, which in turn stimulates cancer cells to activate its adaptive repair pathways through macromolecular response, many of which depend on cellular metabolism (5,52,53). The primary anticancer mechanism of radiotherapy is that radiation can induce different types of lethal damage on DNA. However, the high genomic instability of cancer cells reduces radiation-induced DNA damage before it reaches toxic levels (44). Therefore, an effective DNA damage response is partially determined by the innate radioresistance of cancer cells; further studies are required to focus on how to effectively increase the radiosensitivity of cancer cells when facing different types of radiation by targeting DNA repair pathways (54-56). DNA repair relies on the rich nucleotide pool supported by the active metabolism. These pools afford the essential demands for chromatin modifications and protein activation (57-59).

For single- and double-strand DNA breaks, purine and pyrimidine nucleotide pools are necessary and essential. In fact, experimental studies have demonstrated that an increased nucleotide synthesis rate and more active pathways related to these processes are closely associated with the radioresistance of cancer cells (60,61). Notably, nucleotide metabolism presents context-dependent characteristics in different types of cancer following radiotherapy. The inhibition of GMP resynthesis with mycophenolate mofetil has also been shown to radiosensitize GBMs (62). Glutamine is a precursor for *de novo* nucleotide synthesis; a previous *in vitro* and *in vivo* study demonstrated delayed DNA repair and enhanced radiosensitivity in cancer models followign the knockdown of glutamine synthesis (63). Other pathways involved in nucleotide synthesis in cancer cells include the PPP ribo5-phosphate (R5P) and serine-glycine metabolism during the post-radiation. The involvement of these pathways in promoting radioresistance through DNA repair warrants further investigation (64). Histone methylation and acetylation patterns can regulate DNA repair conditionally (65), although the mechanisms involved remain unclear (66). ATP is associated with protein phosphorylation and the activation of proteins involved in DNA repair (67). Notably, in hepatoma cells, combination therapy with metformin and radiation has been shown to reduce ATP levels in cancer cells and decreases DNA repair capacity, thereby enhancing radiosensitivity (68).

Fatty acids constitute the cornerstone of cell membranes. They are the base of repair on damage induced by radiation. Cells meet their metabolic lipid requirements through *de novo* biosynthesis and uptake from the microenvironment (69). Fatty acid synthase (FASN), a key enzyme among *de novo* palmitate synthase, is associated with cancer radioresistance (70). FASN overexpression is associated with the cancer cell radioresistance, whereas the inhibition of FASN enhances the anticancer effects of radiation (71); however, the precise mechanisms involved remain to be elucidated. FASN may increase intracellular palmitate levels and regulate the expression of PARP1 through the NF-κB pathway (72), thereby supporting radiation resistance through NF-κB-mediated signaling (73).

The dysregulation of cholesterol synthesis is also linked to cellular radioresistance. Cholesterol plays a crucial role in organizing the cell membrane and forming the vascular system, both of them benefit cancer cells following radiation exposure (74). Zoledronic acid, an inhibitor of farnesyl pyrophosphate synthase, sensitizes radiation-resistant pancreatic cancer lines. Conversely, the inhibition of cholesterol synthesis is reportedly associated with the increased survival of patients with lung cancer undergoing radiotherapy (75). These converse results emphasize the functional and environmental specific changes in lipid metabolism that mediate radiation resistance. Apart from the inherent metabolic heterogeneity of cancer cells, the role of lipid metabolism may be determined by the availability of different lipids in the TME following radiotherapy.

## 6. TME metabolism alterations influence radiotherapy outcomes

The complexity of the metabolism of TME components can influence cell-cell interactions, and can even alter the outcomes of diseases. Therefore, understanding the metabolic status of the TME following radiotherapy is essential.

Metabolites provided by the extracellular matrix can promote cancer recovery following radiotherapy (76). In the TME, metabolic productions of larger amounts of non-malignant cells can also promote cancer cell growth in environments that lack nutrients (77). Glutamine from cancer-associated fibroblasts (CAFs) and serine from neurons in the TME can enhance cancer metabolism by supplying ribose-1-phosphate, thereby improving the DNA repair capacity after radiation exposure (78). The TME can also provide lipids to cancer cells, supporting their survival following radiotherapy (79). CAFs have been demonstrated to promote pancreatic and colorectal cancers progression via lipid and branched-chain ketoacid supply (80). Cancer cells can also induce adipocyte fat breakdown, resulting in releasing the fatty acids into the TME. The hypoxic state following radiation therapy reduces lipid synthesis pathways, increasing cancer cell dependence on exogenous lipid uptake (81). Hypoxia induces the expression of HIF, which can facilitate the cancer cells enriching fatty acids, thereby protecting them from radiation-induced ROS (82). Hypoxia also promotes cancer cells to adsorb unsaturated fatty acids by activating serum/glucocorticoid-regulated kinase 1 (SGK1), whereas the inhibition of SGK1 can enhance the effects of radiotherapy (83). Radiation exposure increases intercellular ROS levels in cancers, and substantial ROS levels in the TME are associated with precancerous lesions (45). Through the stabilization of HIF-1α by ROS and the promotion of angiogenesis, the survival of cancer cells is enhanced under radiotherapy. ROS can also promote the activation of CAFs (84,85).

During the post-radiation time, changes in metabolic factors may reprogram the immune activity in the TME, such as the increased production of ROS which can inhibit immune cells (5,52). ROS can affect the phenotype of tumor-associated macrophages (TAMs), promoting a change into a protumor state; ROS can also lead to the recruitment of more Tregs and MDSCs at tumor sites, suppress T-cell activity, and reduce the number of natural killer cells in the TME (86). With elevated levels of lipids in the TME, immune cell function may be altered. For example, excess free fatty acids can lead to lipid accumulation in natural killer cells, thereby reducing their immune capacity. In addition, metabolic interactions between effector T-cells, cancer cells and Tregs can increase lipid accumulation, inducing cellular senescence (69). Effector T-cells and, tumor-promoting Tregs/TAMs have very different energy demands, namely aerobic glycolysis and fatty acid oxidation, respectively. Systemic radiation can lead to lipid accumulation and dysfunction in dendritic cells, thereby inducing a dysfunction in the immune response, which is linked to carcinogenesis (87). As previously mentioned, the extracellular matrix may secrete lipids, and obesity may contribute to elevated lipid levels in the TME (79,80). Increased glycolytic activity following radiation leads to lactate accumulation and TME acidification. High lactate levels inhibit effector T-cell function, promote macrophage differentiation into the immunosuppressed M2 phenotype, and alter monocyte secretion of pro-inflammatory cytokines. Therefore, even though radiation can promote immune cell with higher capacity, metabolic reorganization in cancer cells may contribute to an immunosuppressive environment.

## 7. Effect and regulation of nanodomains on the TME

Tumors are commonly considered as dependent pseudo-organs. In these pseudo-organs, there is a complex interaction between cancer cells and various non-tumor cells, forming a complex network. In this network, cancer cells strive to maintain their malignant proliferation by altering their metabolic properties continuously (88). As is commonly known, cancer cells exhibit an upregulated glycolysis and downregulated oxidative phosphorylation (OXPHOS). However, in a previous study, OXPHOS was reported to be upregulated, even occurring concurrently with a high level of glycolysis (89).

Cancer cells have a high heterogeneity as regards the metabolic phenotype. In cancer cells, some substances, such as glucose, lactate, pyruvate and hydroxybutyrate have a higher metabolic rate than in normal cells, contributing to the complex tumor metabolic ecology. This metabolic heterogeneity and complexity help cancer cells to maintain a dominant position in energy acquisition, whether they utilize ATP, maintain redox balance, or establish metabolic coupling with any other cells (90). Regardless, acquiring energy and essential substances for anabolism determine the fate of any type of cells. Within the same biological system, different cell types compete for energy demands through specific mechanisms. Only those cells that prioritize energy utilization can develop preferentially. The following paragraph discusses innate biological nano-confinements (iBNCs). iBNCs are a proposed concept referring to any naturally occurring structural and functional nanodomains within biological systems.

Nano-confinement refers to the restriction of materials within nanoscale regions. When certain materials, such as some bioactive molecules (such as protein, DNA and RNA) are confined at the nanoscale, they may exhibit a unique behavior distinct from that observed at the macroscale (91). Furthermore, the nano-confinement approach provides a basis for the development of novel cancer therapeutic strategies related to energy and substance utilization (92,93). It is considered that biological nano-confinements widely exist within biological systems, referred to as iBNCs, which play critical roles in tumorigenesis, progression and metastasis via novel mechansisms (94). For instance, CAR-T therapy has exhibited promising outcomes in hematological malignancies. However, in the majority of solid tumors, such as liver, gastric and lung cancer, its effects are limited (95). The higher fluidity of hematological malignancies does not provide a stable environment of the formation and maintenance of iBNCs; therefore, these malignancies often develop resistance to therapeutic interventions, such as chemotherapies and radiotherapies. However, as solid tumors have a relatively stable physical environment, iBNCs can easily be formed. They have the additional capacity to resist drugs/radiation, and ultimately survive from intervention strategies (94).

## 8. Conclusions and future perspectives

Cancer cells can alter and reprogram their metabolic profile in response to the cellular damage induced by radiation. The TME plays a crucial role by supplying precursor substances that support cancer metabolism alterations, presenting potential targets for therapeutic intervention. A key consideration is that iBNCs may help to elucidate the prioritization of energy and substance utilization by cancer cells in solid tumors in hypoxic and nutrition-deprived environments. This may provide further insight into the reason why CAR-T therapies are more effective for hematological cancers than solid tumors, and could explain the synergetic enhancement observed when CAR-T therapy is used in combination with chemotherapy. Further studies are required to focus on validating the therapeutic potential of targeting radiation-induced metabolic reprogramming through preclinical and clinical research, with an emphasis on tumor-type-specific metabolic responses.

## Data Availability

Not applicable.
